# Python in neuroscience

**DOI:** 10.3389/fninf.2015.00011

**Published:** 2015-04-14

**Authors:** Eilif Muller, James A. Bednar, Markus Diesmann, Marc-Oliver Gewaltig, Michael Hines, Andrew P. Davison

**Affiliations:** ^1^Center for Brain Simulation, Ecole Polytechnique Fédérale de LausanneGeneva, Switzerland; ^2^Institute for Adaptive and Neural Computation, University of EdinburghEdinburgh, UK; ^3^Jülich Research Center and Jülich Aachen Research Alliance, Institute of Neuroscience and Medicine (INM-6) and Institute for Advanced Simulation (IAS-6)Jülich, Germany; ^4^Department of Psychiatry, Psychotherapy and Psychosomatics, Medical Faculty, RWTH Aachen UniversityAachen, Germany; ^5^Department of Physics, Faculty 1, RWTH Aachen UniversityAachen, Germany; ^6^Department of Neurobiology, Yale UniversityNew Haven, CT, USA; ^7^Neuroinformatics group Unité de Neurosciences, Information et Complexité, Centre National de la Recherche ScientifiqueGif sur Yvette, France

**Keywords:** python language, software development, scientific computing, interoperability, collaboration

This Research Topic of Frontiers in Neuroinformatics is dedicated to the memory of Rolf Kötter (1961–2010), who was the Frontiers Associate Editor responsible for this Research Topic, and who gave us considerable support and encouragement during the process of conceiving and launching the Topic, and throughout the reviewing process.

Computation is becoming essential across all sciences, for data acquisition and analysis, automation, and hypothesis testing via modeling and simulation. As a consequence, software development is becoming a critical scientific activity. Training of scientists in programming, software development, and computational thinking (Wilson, 2006), choice of tools, community-building and interoperability are all issues that should be addressed, if we wish to accelerate scientific progress while maintaining standards of correctness and reproducibility.

The Python programming language in particular has seen a surge in popularity across the sciences, for reasons which include its readability, modularity, and large standard library. The use of Python as a scientific programming language began to increase with the development of numerical libraries for optimized operations on large arrays in the late 1990s, in which an important development was the merging of the competing Numeric and Numarray packages in 2006 to form NumPy (Oliphant, 2007). As Python and NumPy have gained traction in a given scientific domain, we have seen the emergence of domain-specific ecosystems of open-source Python software developed by scientists. It became clear to us in 2007 that we were on the cusp of an emerging *Python in neuroscience* ecosystem, particularly in computational neuroscience and neuroimaging, but also in electrophysiological data analysis and in psychophysics.

Two major strengths of Python are its modularity and ability to easily “glue” together different programming languages, which together facilitate the interaction of modular components and their composition into larger systems. This focus on reusable components, which has proven its value in commercial and open-source software development (Brooks, 1987), is, we contend, essential for scientific computing in neuroscience, if we are to cope with the increasingly large amounts of data being produced in experimental labs, and if we wish to understand and model the brain in all its complexity.

We therefore felt that it was timely and important to raise awareness of the emerging Python in Neuroscience software ecosystem amongst researchers developing Python-based tools, but also in the larger neuroscience community.

Our goals were several-fold:

- establish a critical mass for Python use and development in the eyes of the community;
- encourage interoperability and collaboration between developers;
- expose neuroscientists to the new Python-based tools now available.

From this was born the idea for a Research Topic in *Frontiers in Neuroinformatics* on “Python in Neuroscience” to showcase those projects we were aware of, and to give exposure to projects of which we were not aware. Although it may seem strange at first glance to center a Research Topic around a tool, rather than around a scientific problem, we feel it is justified by the increasingly critical role of scientific programming in neuroscience research, and by the particular strengths of the Python language and the broader Python scientific computing ecosystem.

Collected in this Research Topic are 24 articles describing some ways in which neuroscience researchers around the world are turning to the Python programming language to get their job done faster and more efficiently.

## Overview of the Research Topic

We will now briefly summarize the 24 articles in the Research Topic, drawing out common themes.

Both Southey et al. (2008) and Yanashima et al. (2009) use Python for bioinformatics applications, but in very different areas. Yanashima et al. have developed a Python package for graph-theoretical analysis of biomolecular networks, BioNetpy, and employed it to investigate protein networks associated with Alzheimer's disease. Southey et al.'s study demonstrates the wide breadth of application of Python, and the large number of high quality scientific libraries available, combining existing tools for bioinformatics, machine learning and web development to build an integrated pipeline for identification of prohormone precursors and prediction of prohormone cleavage sites.

Jurica and van Leeuwen (2009) address the needs of scientists who already have significant amounts of code written in MATLAB® and who wish to transfer this to Python. They present OMPC, which uses syntax adaptation and emulation to allow transparent import of existing MATLAB® functions into Python programs.

Three articles reported on new tools in the domain of neuroimaging. Hanke et al. (2009) report on PyMVPA, a Python framework for machine learning-based data analysis, and its application to analysis of fMRI, EEG, MEG, and extracellular electrophysiology recordings. Gouws et al. (2009) describe DataViewer3D, a Python application for displaying and integrating data from multiple neuroimaging modalities, showcasing Python's abilities to easily interface with libraries written in other languages, such as C++, and to integrate them into user-friendly systems. Strangman et al. (2009) emphasize the advantages of Python for “*swift prototyping followed by efficient transition to stable production systems*” in their description of NinPy, a toolkit for near-infrared neuroimaging.

Zito et al. (2009) and Ince et al. (2009) both report on the use of Python for general purpose data analysis, with a focus on machine learning and information theory respectively. Zito et al. have developed MDP, the Modular toolkit for Data Processing, a collection of computationally efficient data analysis modules that can be combined into complex pipelines. MDP was originally developed for theoretical research in neuroscience, but has broad application in general scientific data analysis and in teaching. Ince et al. (2009) describe the use of Python for information-theoretic analysis of neuroscience data, outlining algorithmic, statistical and numerical challenges in the application of information theory in neuroscience, and explaining how the use of Python has significantly improved the speed and domain of applicability of the algorithms, allowing more ambitious analyses of more complex data sets. Their code is available as an open-source package, pyEntropy.

Three articles report on tools for visual stimulus generation, for use in visual neurophysiology and psychophysics experiments. Straw (2008) describes VisionEgg, while Peirce (2009) presents PsychoPy, both of which are easy-to-use and easy-to-install applications that make use of OpenGL to generate temporally and spatially precise, arbitrarily complex visual stimulation protocols. Python is used to provide a simple, intuitive interface to the underlying graphics libraries, to provide a graphical user interface, and to interface with external hardware. PsychoPy can also generate and deliver auditory stimuli. Spacek et al. (2009) also report on a Python library for visual stimulus generation, as part of a toolkit for the acquisition and analysis of highly parallel electrophysiological recordings from cat and rat visual cortex. The other two components in the toolkit are for electrophysiological waveform visualization and spike sorting; and for spike train and stimulus analysis. The authors note “*The requirements and solutions for these projects differed greatly, yet we found Python to be well suited for all three.”*

Also in the domain of electrophysiology, Garcia and Fourcaud-Trocmé (2009) describe OpenElectrophy, an application for efficient storage and analysis of large electrophysiology datasets, which includes a graphical user interface for interactive visualization and exploration and a library of analysis routines, including several spike-sorting methods.

By far the largest contribution to the Research Topic came from the field of modeling and simulation, with 12 articles on the topic. Nine of these articles present neuroscience simulation environments with Python scripting interfaces. In most cases, the Python interface was added to an existing simulator written in a compiled language such as C++. This was the case for NEURON (Hines et al., 2009), NEST (Eppler et al., 2009), PCSIM (Pecevski et al., 2009), Nengo (Stewart et al., 2009), MOOSE (Ray and Bhalla, 2008), STEPS (Wils and De Schutter, 2009) and NCS (Drewes et al., 2009). However, as the articles by Goodman and Brette (2008) on the Brian simulator and Bednar (2009) on the Topographica simulator demonstrate, it is also possible to develop new simulation environments purely in Python, making use of the vectorization techniques available in the underlying NumPy package to obtain computational efficiency. The range of modeling domains of these simulators is wide, from stochastic simulation of coupled reaction-diffusion systems (STEPS), through simulation of morphologically detailed neurons and networks (NEURON, MOOSE), highly-efficient large-scale networks of spiking point neurons (NEST, PCSIM, NCS, Brian) to population coding or point-neuron models of large brain regions (Nengo, Topographica). Note that although we have categorized each simulator by its main area of application, most of these tools support modeling at a range of scales and levels of detail: Bednar (2009), for example, describes the integration of a spiking NEST simulation as one component in a Topographica simulation.

The addition of Python interfaces to such a large number of widely used simulation environments suggested a huge opportunity to enhance interoperability between different simulators, making use of the common scripting language, which in turn has the potential to enhance the transfer of technology, knowledge and models between users of the different simulators, and to promote model reuse. Davison et al. (2009a) describe PyNN, a common Python interface to multiple simulators, which enables the same modeling and simulation script to be run on any supported simulator without modification. At the time of writing, PyNN supports NEURON, NEST, PCSIM and Brian, with MOOSE support under development. The existence of such a common “meta-simulator” then makes it much easier for scientists developing new, hardware-based approaches to neural simulation to engage with the computational neuroscience community, as evidenced by the article by Brüderle et al. (2009) on interfacing a novel neuromorphic hardware system with PyNN.

Finally, Fox et al. (2009) describe the possibilities when one is not limited to a single simulator, but can use Python to integrate multiple models into a brain-wide system. In their development of an integrated basal ganglia-hippocampal formation model for spatial navigation and its embodiment in a simulated robotic environment, Fox et al. found that Python offers “*a significant reduction in development time, without a corresponding significant increase in execution time*.”

It is important to note that most or all of the Python tools and libraries described in the Research Topic are open source and hence free to download, use and extend.

## Discussion

This editorial is being written 6 years after the first articles in the Research Topic were published. It is with the benefit of considerable hindsight, therefore, that we can confidently say that our goals in launching this Research Topic—to establish a critical mass for Python use and development in the eyes of the community and to encourage interoperability and collaboration between developers—have been met or exceeded.

The average number of citations per article for the Research Topic as a whole is 54, or approximately 9 per year, using figures from Google Scholar. Although citation counts from Google Scholar tend to be higher than those from Journal Citation Reports so the numbers are not directly comparable, this compares favorably with the impact factors of well respected journals such as Journal of Neuroscience or PLoS Computational Biology. Some of the articles were much more highly cited, with three of them being cited more than 20 times per year, on average, over the period. Four of the articles were chosen to “climb the tier” in the Frontiers system, and were followed up by Focused Review articles in Frontiers in Neuroscience (Davison et al., 2009b; Goodman and Brette, 2009; Hanke et al., 2010; Ince et al., 2010), another was the subject of a commentary (Einevoll, 2009).

Concerning the goals of interoperability and collaboration, several articles in a follow-up volume *Python in Neuroscience II* attest to the degree to which the developers of different tools have worked together, and prioritized interoperability in recent years. For example, the developers of OpenElectrophy (Garcia and Fourcaud-Trocmé, 2009) and the community around PyNN (Davison et al., 2009a) formed the nucleus of an effort to develop a baseline Python representation for electrophysiology data, which resulted in the Neo project, reported in the *Python in Neuroscience II* Research Topic (Garcia et al., 2014) together with two of the several projects which build on Neo (Pröpper and Obermayer, 2013; Sobolev et al., 2014). A new workflow system for computational neuroscience, Mozaik (Antolík and Davison, 2013) builds on both PyNN and Topographica (Bednar, 2009). PyNEST (Eppler et al., 2009) and PyNN developers collaborated with the INCF to improve the interoperability between these tools (Djurfeldt et al., 2014) when using the Connection Set Algebra (Djurfeldt, 2012). Finally, a number of tools have been built on the Python interface to NEURON (Hines et al., 2009), including morphforge (Hull and Willshaw, 2014) and LFPy (Lindén et al., 2014).

Observing the rapid growth in adoption of Python in neuroscience over the last 6 years, which appears to continue to accelerate, it is clear that Python is here to stay, which augurs well for the growth, productivity, and rigor of computational methods in neuroscience.

### Conflict of interest statement

The authors declare that the research was conducted in the absence of any commercial or financial relationships that could be construed as a potential conflict of interest.
